# Cardiovascular Comorbidities Do Not Impact Prostate Artery Embolisation (PAE) Outcomes: Retrospective Analysis of the National UK-ROPE Registry

**DOI:** 10.1007/s00270-023-03608-6

**Published:** 2023-11-27

**Authors:** Ganesh Vigneswaran, Drew Maclean, Neel Doshi, Mark Harris, Timothy J. C. Bryant, Nigel C. Hacking, Bhaskar Somani, Sachin Modi

**Affiliations:** 1 Department of Interventional Radiology, University Hospital Southampton, Southampton, UK; 2 Department of Urology, University Hospital Southampton, Southampton, UK; 3 Cancer Sciences, University of Southampton, Southampton, UK

**Keywords:** Prostate artery embolisation (PAE), Benign prostatic hyperplasia (BPH), Lower urinary tract symptoms (LUTS), Cardiovascular risk, Hypertension, Diabetes, Smoking

## Abstract

**Purpose:**

Prostate artery embolisation (PAE) is a key treatment for the management of symptomatic benign prostatic hyperplasia (BPH). Common cardiovascular risk factors might be associated with suboptimal outcomes and thus influence patient treatment selection. The aim of the study was to evaluate whether cardiovascular comorbidities affect PAE outcomes.

**Methods:**

Retrospective subset analysis of the UK Registry of Prostate Artery Embolisation (UK-ROPE) database was performed with patients who had a full documented past medical histories including hypertension, diabetes, coronary artery disease (CAD), diabetes and smoking status as well as international prostate symptom score (IPSS) at baseline and at 12 months. Multiple regression was performed to assess for any significant predictors.

**Results:**

Comorbidity data were available for 100/216 patients (mean age 65.8 ± 6.4 years), baseline IPSS 20.9 ± 7.0). Regression analysis revealed that the presence of hypertension (53.7% IPSS reduction vs. absence 51.4%, *p* = 0.94), diabetes (52.6% vs. absence 52.1%, *p* = 0.6), CAD (59.2% vs. absence 51.4%, *p* = 0.95), no comorbidities (49.8% vs. any comorbidity present 55.3%, *p* = 0.66), smoking status (non-smoker, 52.6%, current smoker, 61.5%, ex-smoker, 49.8%, *p* > 0.05), age (*p* = 0.52) and baseline Qmax (*p* = 0.41) did not significantly impact IPSS reduction at 12 months post-PAE. Baseline prostate volume significantly influenced IPSS reduction (≥ 80 cc prostates, 58.9% vs. < 80 cc prostates 43.2%, *p* < 0.05).

**Conclusion:**

The presence of cardiovascular comorbidities/smoking history does not appear to significantly impact PAE symptom score outcomes at 12 months post procedure. Our findings suggest that if the prostatic artery can be accessed, then clinical success is comparable to those without cardiovascular comorbidities.

**Graphical Abstract:**

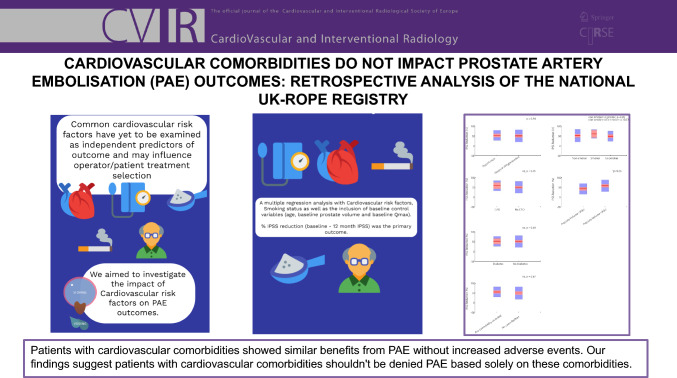

## Introduction

Patient selection remains one of the key issues surrounding prostate artery embolisation (PAE) [1] for treatment of benign prostatic hyperplasia (BPH). Identification of patients with the best chance of symptom improvement versus candidates who are unlikely to attain therapeutic success from PAE could serve as a vital strategy for averting superfluous interventions and reducing incidence of side-effects. While predictors of outcomes following PAE have been established, studies thus far have been more focused on technical aspects of the procedure such as evaluating embolic agent type, embolic size and number of arteries embolised [2–7].

Transurethral resection of prostate (TURP) and other transurethral approaches typically require a general or regional anaesthetic [8]. This is in contrast to the minimally invasive technique of PAE requiring only local anaesthetic ± sedation [9]. In this respect, patients with comorbidities (particularly cardiovascular comorbidities such as coronary artery disease, hypertension, type II diabetes, stroke) that have a higher anaesthetic risk, can now be offered a less invasive and safer alternative with PAE [10].

However, there are concerns around performing PAE in patients with cardiovascular comorbidities. A recent retrospective study found that cardiovascular comorbidities was a single independent variable inversely associated with PAE clinical success [11]. Additionally as most cardiovascular comorbidities are associated with atherosclerosis [12], there are theoretical concerns around collateral circulations reducing therapeutic efficacy of the procedure and/ or plaque associated turbulent flow increasing risk of non-target embolisation [4]. As such, evaluating whether cardiovascular risk factors are independent predictors of PAE outcomes would help stratification of patients and guide clinical decision making.

The aim of our study is to evaluate the impact of cardiovascular risk factors on PAE outcomes.

## Materials and Methods

We carried out a retrospective analysis of a prospectively collected multicentre (17 centres) PAE cohort from the UK-ROPE database [2, 3, 13, 14]. Details of the original study, including the original inclusion and exclusion criteria, have previously described extensively [3]. For this specific analysis, we also excluded incomplete cardiovascular comorbidity data at baseline, absent IPSS score at baseline or patients who did not complete 12 months clinical follow-up. The primary outcome was normalised reduction in International Prostate Symptom Score (IPSS) at 12 months follow-up for each association of the presence or absence of several cardiovascular risk factors (including hypertension, coronary artery disease (CAD), diabetes and smoking status). Study size included all eligible patients from the database.

## Statistical Analysis

A multiple regression analysis was performed with cardiovascular risk factors (described above) as well as baseline control variables (age, baseline prostate volume and baseline Qmax (maximum flow rate) where present). As per previous studies, prostate volume data were dichotomised into < 80 cc and > = 80 cc groups. Normalised IPSS reduction; ((baseline IPSS—12 month IPSS)/baseline IPSS) * 100) was the primary outcome. A significance level of 0.05 was used for analysis. Figures include data means (solid line) 1.96 × standard error of the mean (95% confidence interval) in red and 1 standard deviation in blue. All statistical tests were performed using MATLAB 2021a (MathWorks, USA).

## Results

Of 216 patients recruited to the initial registry, 100 patients were eligible for inclusion (95 did not attend 12-month follow-up and 21 had incomplete risk factor documentation). The baseline parameters for this selected cohort are shown in Table 1 alongside outcomes**.**

**Table 1:** Baseline characteristics and IPSS outcomes of variables and comorbidities used in multiple regression analysis

Variables	*N* or mean (SD)	Mean normalised IPSS % reduction (SD)	*p*-value
Baseline IPSS	20.9 (7.0)	n/a	n/a
Baseline prostate volume (cc)	99.5 (55.6) [≥ 80 cc (n = 57) versus < 80 cc (n = 43)]	58.9 (29.9) 43.2 (27.6)	0.046
Baseline Qmax (ml/s)	8.5 (5.0)	n/a	0.41
Hypertension	Presence: *n* = 33 Absence: *n* = 67	53.7 (25.9) 51.4 (26.2)	0.94
Coronary artery disease (CAD)	Presence: *n*= 10 Absence: *n* = 90	59.2 (26.2) 51.4 (30.2)	0.95
Diabetes	Presence: *n* = 13 Absence: *n* = 87	52.6 (25.8) 52.1 (30.5)	0.60
Any comorbidities	Yes: *n* = 43 No: *n* = 57	55.3 (26) 49.8 (32.5)	0.66
Age (years)	65.8 (6.4)		0.52
Smoking status	Current Smoker *n* = 6/100 Ex-smoker *n* = 35/100 Non-smoker *n* = 59/100	61.5 (23.1) 49.8 (25.9) 52.6 (32.7)	0.82 0.63

The *p*-values are attributable to multiple regression analysis

**Fig. 1:**
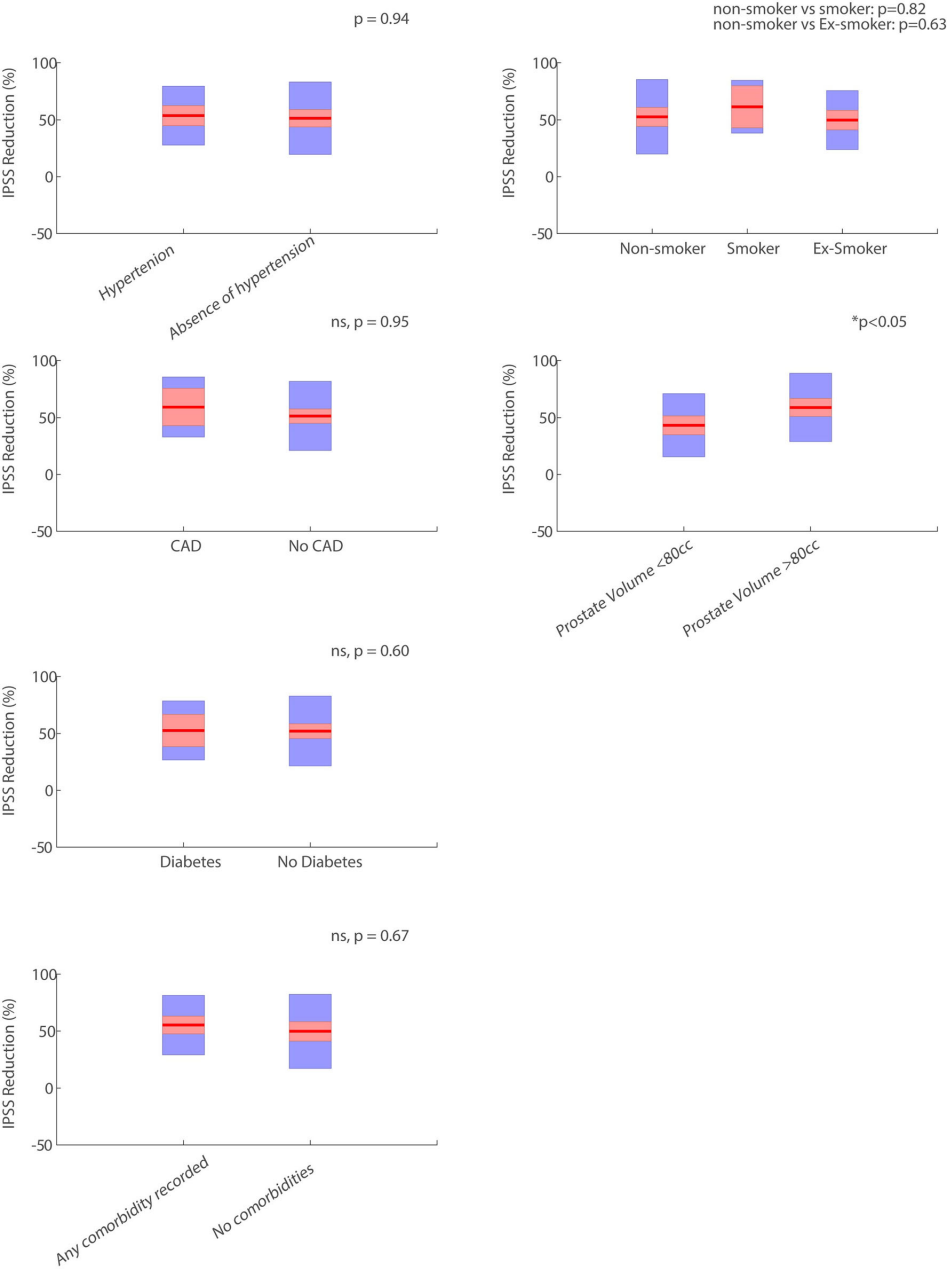
Presence or absence of risk factors versus IPSS reduction (%). The mean is plotted as a horizontal red line. Ninety-five percentage of confidence intervals are plotted in shaded red as well as standard deviation shaded in blue

Our results demonstrated that none of the evaluated cardiovascular comorbidities, smoking status, age or baseline Qmax significantly impacted normalised IPSS reduction at 12 months post-PAE as demonstrated in Table 1 and Fig. 1. We found baseline prostate volume was the single independent variable that influenced normalised IPSS reduction, with larger prostates demonstrating greater normalised IPSS reduction. There were no significant adverse complications in patients with cardiovascular comorbidities (Table 2).

**Table 2:** Table to demonstrate complication rates in patients with and without any assessed cardiovascular comorbidities

	Presence of any assessed cardiovascular comorbidity	No cardiovascular comorbidity
Total assessed	*N* = 43	*N* = 57
Significant complications at procedure (Clavien–Dindo classification ≥ 3)	*N* = 0	*N* = 0
Minor complications at procedure (Clavien–Dindo classification < 3)	*N* = 1 (2.3%)	*N* = 4 (7.0%)
Patient reported complications by 30 days	*N* = 3 (7.0%) including haematuria and haematospermia	*N* = 8 (14.0%) including haematuria, haematospermia and urinary tract infection

## Discussion

Our analysis indicates that patients with cardiovascular comorbidities including diabetes, coronary artery disease and hypertension receive similar benefits from PAE at 12 months without any increase in adverse events. This study supports previous literature promoting PAE for those unfit for general anaesthesia-based surgeries [8]. As PAE is typically performed in elderly patients, who frequently have cardiovascular comorbidities (43% in our cohort), our results show promising clinical outcomes despite these comorbidities. Our findings differ from a study by Frandon et al., which found an inverse association between cardiovascular comorbidities and PAE success [11]. However, the study defined clinical success as IPSS reduction at 3 months or successful removal of an indwelling catheter, while we evaluated IPSS reduction at 12 months in a non-catheterised population. Furthermore, there were differences in defining cardiovascular comorbidities where Frandon et al. specifically evaluated presence of lower limb arterial disease, a limitation of our analysis, but did not consider smoking status. Although PAE clinical success typically manifests within 3 months post procedure [11], previous literature has suggested that in some patients it can take up to 6 months to manifest [15].

While our research provides positive insights, there are notable limitations of this study. Although we included many significant comorbidities, we did not evaluate all cardiovascular conditions as the UK-ROPE was not conceived to answer this as the primary clinical question [3]. As such not all significant cardiovascular comorbidities (e.g. stroke) or drivers of atherosclerosis (e.g. chronic kidney disease) have been evaluated. Furthermore, from a technical perspective, atherosclerosis in the internal iliac arteries also pose challenges for performing the procedure, which we did not specifically consider [3, 16]. We also acknowledge the potential risk of non-reporting bias in terms of follow-up (only available in 100 out of 216 patients).

There remains a limited body of literature addressing the impact of cardiovascular comorbidities on the outcomes of PAE, and a solitary study cautioning against its therapeutic efficacy. Our study provides evidence to the contrary and is based on a prospectively collected multicentre data with specific secondary research end point to evaluate the presence of common comorbidities on PAE clinical success.

## Conclusion

Our study findings cautiously support the use of PAE in patients with cardiovascular comorbidities. These findings suggest that if the prostatic artery can be accessed, then clinical success is comparable to those without cardiovascular comorbidities and thus should not be refused treatment on these grounds alone.
